# Felbamate but not phenytoin or gabapentin reduces glutamate release by blocking presynaptic NMDA receptors in the entorhinal cortex

**DOI:** 10.1016/j.eplepsyres.2007.09.005

**Published:** 2007-12

**Authors:** Jian Yang, Caroline Wetterstrand, Roland S.G. Jones

**Affiliations:** Department of Pharmacy and Pharmacology, University of Bath, Claverton Down, Bath BA2 7AY, UK

**Keywords:** Entorhinal cortex, Presynaptic NMDA receptors, Glutamate release, Phenytoin, Felbamate, Gabapentin

## Abstract

We have shown that a number of anticonvulsant drugs can reduce glutamate release at synapses in the rat entorhinal cortex (EC) *in vitro*. We have also shown that presynaptic NMDA receptors (NMDAr) tonically facilitate glutamate release at these synapses. In the present study we determined whether, phenytoin, gabapentin and felbamate may reduce glutamate release by blocking the presynaptic NMDAr. Whole cell patch clamp recordings of spontaneous excitatory postsynaptic currents (sEPSCs) were used as a monitor of presynaptic glutamate release. Postsynaptic NMDAr were blocked with internal dialysis with an NMDAr channel blocker. The antagonist, 2-AP5, reduced the frequency of sEPSCs by blocking the presynaptic facilitatory NMDAr, but did not occlude a reduction in sEPSC frequency by gabapentin or phenytoin. Felbamate also reduced sEPSC frequency, but this effect was occluded by prior application of 2-AP5. Thus, whilst all three drugs can reduce glutamate release, only the action of felbamate seems to be due to interaction with presynaptic NMDAr.

## Introduction

Experiments in this laboratory have shown that the anticonvulsant drugs phenytoin, lamotrigine, gabapentin, pregabalin and valproate can reduce the spontaneous release of glutamate from excitatory terminals in the rat entorhinal cortex (EC) *in vitro* (Cunningham and Jones, 2000; Cunningham et al., 2000, 2003, 2004). The effect of valproate alone appeared to depend on blockade of voltage-gated sodium channels (VGSC; Cunningham et al., 2003), whereas the reduction in release elicited by the other drugs was independent of any action on sodium channels. Gabapentin and pregabalin appeared to act partly via an action on voltage-gated calcium channels (VGCC), and partly via an unknown mechanism (Cunningham et al., 2004).

We have also previously shown that glutamate release in the EC is tonically facilitated by ambient glutamate acting via presynaptic NMDA autoreceptors (Berretta and Jones, 1996a; Woodhall et al., 2001; Yang et al., 2006), an effect confirmed in visual cortex (Li and Han, 2007). Recently, it has been suggested that gabapentin may interact with presynaptic NMDA receptors (NMDAr) in the hippocampus (Suarez et al., 2005). Furthermore, pregabilin has been suggested to reduce GABA and, possibly, glutamate release in hippocampal cultures via an interaction with presynaptic NMDAr (Micheva et al., 2006). There is some evidence, albeit scant, to suggest that phenytoin may block NMDAr (e.g. Wamil and McLean, 1993; Naskar et al., 2002), and that it may reduce NMDAr-stimulated monoamine transmitter release (Sethy and Sage, 1992; Brown et al., 1994). Thus, we have considered the possibility that the reduction of glutamate release by anticonvulsants in the EC may depend on blockade of presynaptic facilitatory NMDAr.

To investigate this possibility we monitored glutamate release in rat EC slices by recording spontaneous excitatory postsynaptic currents (sEPSCs) mediated by AMPA receptors (AMPAr) using whole-cell voltage clamp recordings. When postsynaptic NMDAr are blocked by inclusion of MK-801 in the patch pipette, competitive NMDAr antagonists reduce the frequency (but not amplitude or kinetics) of sEPSCs, by blocking the tonic presynaptic facilitation of release (Berretta and Jones, 1996a; Woodhall et al., 2001; Yang et al., 2006; Li and Han, 2007). Thus, we determined whether the reduction in frequency of sEPSCs by phenytoin and gabapentin (Cunningham et al., 2000, 2004) could be occluded by prior blockade of presynaptic NMDAr. We compared the effects of these two drugs to those of felbamate, a second-generation anticonvulsant. Felbamate has consistently been shown to block NMDAr-mediated currents (e.g. Harty and Rogawski, 2000; Kuo et al., 2004), so could also potentially alter glutamate release via an action at presynaptic NMDAr.

The results suggest that neither the VGSC-independent reduction of glutamate release by phenytoin, nor the VGCC-independent effect of gabapentin, are due to blockade of the presynaptic NMDAr. However, felbamate does appear to reduce release via presynaptic NMDAr blockade, and this could be a factor in its anticonvulsant effect.

## Methods

Combined entorhinal-hippocampal slices were prepared from male Wistar rats, as previously described (Jones and Heinemann, 1988). Rats were killed by cervical dislocation. They were decapitated and the brain was rapidly removed and immersed in oxygenated artificial cerebrospinal fluid (ACSF) chilled to 4 °C. Slices (450 μm) were cut using a Vibroslice, and stored in ACSF bubbled with 95% O_2_/5% CO_2_, at room temperature. Following recovery for at least 1 h, individual slices were transferred to a recording chamber mounted on the stage of a Zeiss Axioskop FS microscope. The chamber was perfused (2 ml/min) with oxygenated ACSF (pH 7.4) at 30–32 °C. The ACSF contained (in mM): NaCl (126), KCl (3.25), NaH_2_PO_4_ (1.25), NaHCO_3_ (24), MgSO_4_ (2), CaCl_2_ (2), and d-glucose (10). Neurones were visualized using differential interference contrast optics and an infrared video camera.

Patch pipettes (1–4 MΩ) were pulled from borosilicate glass on a Flaming/Brown microelectrode puller. Pipettes were filled with a solution containing (in mM): Cs-gluconate (100), HEPES (40), QX-314 (1), EGTA (0.6), NaCl (4), MgCl_2_ (5), TEA-Cl (1), ATP-Na (4), GTP-Na (0.3), MK-801 (1). The solution was adjusted to 275 mOsmol by dilution, and set to pH 7.3 with CsOH. Whole cell voltage clamp recordings were made from neurones in layer V of the medial division of the EC, using an Axopatch 200B amplifier. Using this pipette solution and with membrane potential clamped at −60 mV, neurones displayed sEPSCs. The open channel blocker, MK-801, was included in the patch pipette in order to block postsynaptic NMDAr in the recorded neurone. To facilitate this blockade, neurones were depolarised to −10 mV for 10 s at intervals (every 20 s) during a 10 min period following breakthrough to whole-cell access. Using this approach NMDAr mediated EPSCs are rapidly abolished.

**Figure 1:**
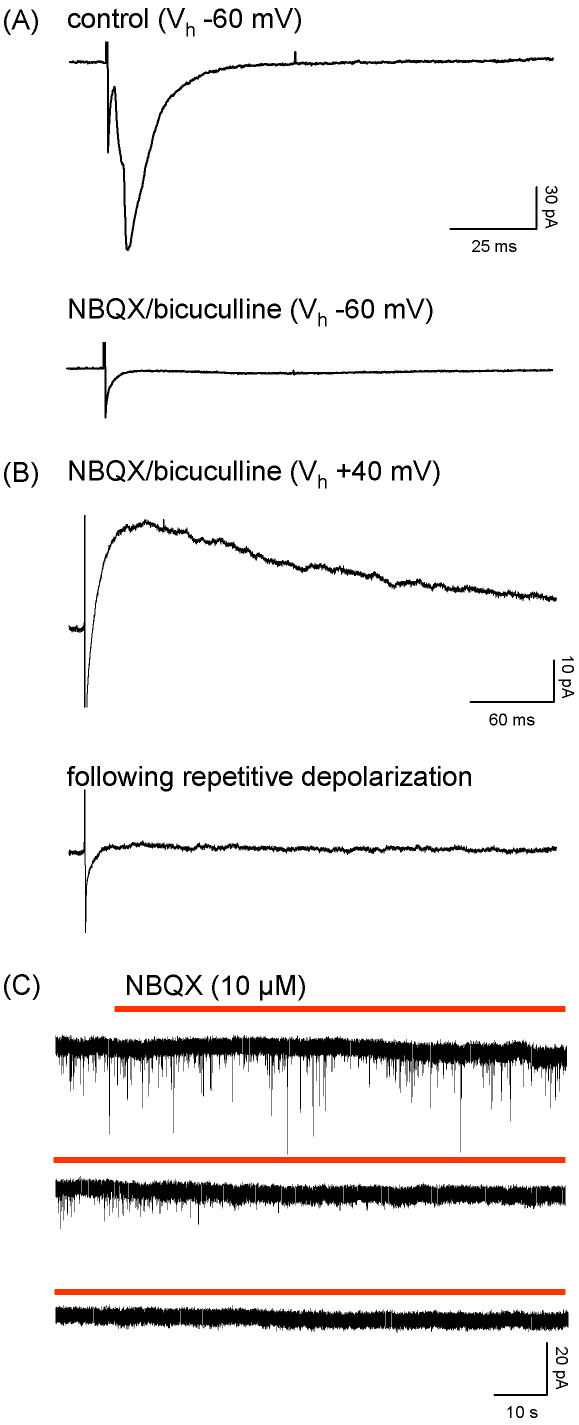
Intracellular MK-801 blocks postsynaptic NMDAr. (A) The traces are voltage clamp recordings of eEPSCs in a layer V neurone (at least eight responses averaged) evoked by electrical stimulation of afferent pathways at 0.05 Hz. The large eEPSC seen at a holding potential of −60 mV, is replaced by a weak slow eEPSC during perfusion with NBQX (10 μM) and bicuculline (30 μM). (B) Shifting the holding potential to +40 mV revealed a large slow eEPSC that was subsequently abolished by a period of repetitive depolarizing steps from −60 to −10 mV over a period of 5 min. (C) Whole cell voltage clamp records of sEPSCs in a different layer V neurone. The three traces are continuous. The patch pipette contained MK-801, and prior to the recordings illustrated, the neurone had been subject to a series of depolarizing voltage steps (as described above and in the text) for a period of 10 min. Addition of NBQX to the perfusion medium (during red bar) rapidly abolished all sEPSCs.

The use of intracellular MK-801 to block postsynaptic responses has been described in detail by us previously (Berretta and Jones, 1996a; Woodhall et al., 2001; Yang et al., 2006). Fig. 1 shows an experiment that confirms the ability of MK-801 to block postsynaptic NMDAr. In this case, EPSCs were evoked (eEPSC) by electrical stimulation in layer V of the lateral EC, with MK-801 in the patch pipette, and at a holding potential of −60 mV (Fig. 1A). Blockade of AMPA and GABA receptors left a small shallow eEPSC. When the holding potential was changed to +40 mV (Fig. 1B), this was revealed as a large slow NMDAr mediated eEPSC. The holding potential was then stepped repetitively from −60 to −10 mV (for just 5 min in this case), and subsequent stimulation at +40 mV showed that the eEPSC was now abolished. This approach has become a widely accepted means of selectively blocking postsynaptic NMDAr, leaving presynaptic receptors intact, and has been used successfully by a number of other groups in cortical, hippocampal and amygdala neurones (e.g. Sjostrom et al., 2003; Massey et al., 2004; Samson and Pare, 2005; Bender et al., 2006; Jourdain et al., 2007; Li and Han, 2007).

Even when postsynaptic receptors are not blocked with MK-801, spontaneous events mediated solely by NMDAr are very infrequent, and there is only a minor contribution of NMDAr to the decay phase of sEPSCs (Berretta and Jones, 1996b). In these circumstances, sEPSCs are abolished by bath application of NBQX and 2-AP5 (e.g. Stacey et al., 2002). Fig. 1C shows recordings of sEPSCs in one neurone and confirms that with postsynaptic NMDAr blocked with intracellular MK-801, addition of an AMPAr antagonist abolishes all spontaneous currents. It should be stressed that all the recordings presented in this paper were conducted with postsynaptic NMDAr blocked and under these experimental conditions, sEPSCs are mediated by glutamate acting at AMPA receptors (Berretta and Jones, 1996b; Woodhall et al., 2001). In other experiments in this laboratory (Chamberlain, S.E.L., Jones, R.S.G.) we have shown that kainate receptors can mediate postsynaptic excitatory responses in the EC, but these receptors are not activated by spontaneously released glutamate. Thus, sEPSCs in our recordings are mediated solely by AMPAr.

The experimental protocols required patch clamp recordings for periods of up to 60 min, so we had to consider the possibility that changes in sEPSCs might be complicated by run-down of events. However, the inclusion of ATP-Na and GTP-Na largely precluded this, and we have previously made similar long duration patch clamp recordings of sEPSCs in many studies without any problems regarding stability (e.g. Berretta and Jones, 1996a,b; Cunningham et al., 2000, 2003, 2004; Cunningham and Jones, 2000; Evans et al., 2001; Woodhall et al., 2001; Stacey et al., 2002; Yang et al., 2006). Series resistance compensation was not employed in the present experiments, but access resistance (10–30 MΩ) was monitored at regular intervals throughout and neurones were discarded from analysis if it changed by more than ±10%. Liquid junction potentials were estimated using the calculator of pClamp 8 software, and compensated for in the holding potentials.

After gaining whole cell access, and allowing time for MK-801 to diffuse into the neurone and block the postsynaptic NMDAr (with repeated depolarizations), the neurone was then allowed to stabilize for a further control period of not less than 10 min after which sEPSCs were recorded. 2-AP5 was then added to the perfusion medium for a period of 20 min before further addition of an anticonvulsant and perfusion of both drugs for a further 20 min. In a second series of experiments, the drugs were perfused in reverse order. sEPSCs were recorded throughout and analysis periods confined to the final 5 min of perfusion with one, and then both drugs.

Data were recorded to computer hard disk using Axoscope software. Minianalysis (Synaptosoft, U.S.A.) was used for analysis of sEPSCs off-line. sEPSCs were detected automatically using a threshold-crossing algorithm. Threshold varied from neurone to neurone, but was always maintained at a constant level in each individual recording. 200 sEPSCs were sampled during a continuous recording period for each neurone under each condition. The populations of events sampled were mixed, and represent both activity-dependent and activity-independent miniature EPSCs (mEPSCs) from multiple terminals on the same neurones, although the majority of events (around 60–70%) are mEPSCs (see Berretta and Jones, 1996b). No attempt was made to separate these in the present study, but we have previously shown that presynaptic NMDAr can facilitate both forms of release (Berretta and Jones, 1996a,b; Woodhall et al., 2001), and also that phenytoin and gabapentin depress the frequency of both sEPSCs, and mEPSCs recorded in the presence of TTX (Cunningham et al., 2000, 2004). To compare pooled data under control and drug conditions we determined mean inter-event interval (IEI), the frequency derived from the IEI, amplitude, and rise and decay times for sEPSCs in each cell. A paired *t*-test was used to compare mean amplitudes and frequencies, rise and decay times, and the non-parametric Kolmogorov–Smirnoff (KS) test to assess the significance of shifts in cumulative probability distributions of IEI. All error values stated in the text refer to standard error of the mean.

The following drugs were used: 2-AP5 (d,l-2-amino-5-phosphonovalerate; Tocris, UK); phenytoin sodium (Sigma, UK); gabapentin (1-(aminomethyl)cycloheaneacetic acid; a gift from Pfizer, Global Research & Development, Ann Arbor, Michigan); felbamate (2-phenyl-1,3-propanediol dicarbamate; Tocris, UK).

## Results

Experiments were performed on a total of 21 neurones in layer V of the medial EC. sEPSCs in control recordings had a mean IEI of 291 ± 44 ms (equating to a frequency of 5.3 ± 0.7 Hz), an amplitude of 12.0 ± 0.9 pA, and rise (10–90%) and decay times (60%) of 1.9 ± 0.1 and 5.8 ± 0.9 ms, respectively.

### Phenytoin

**Figure 2:**
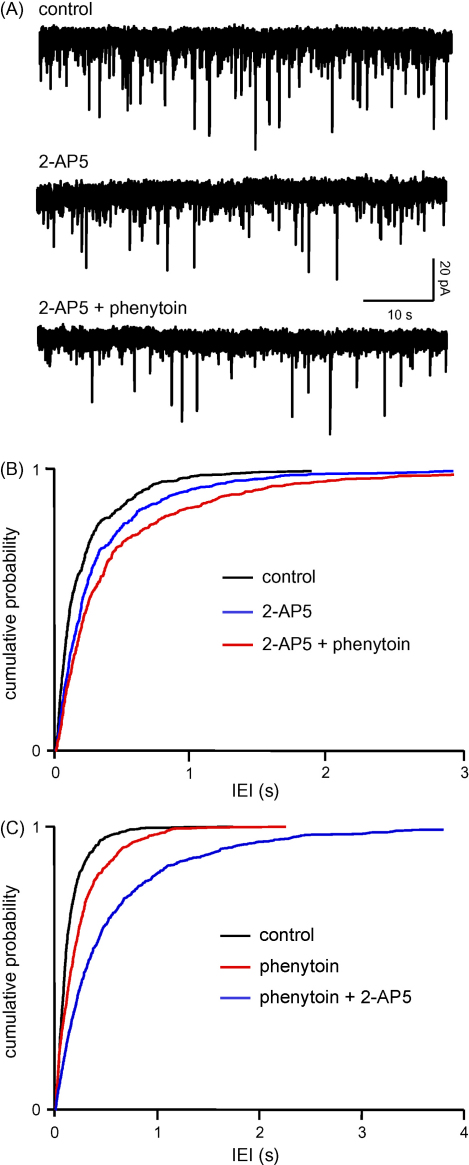
Phenytoin does not act via presynaptic NMDAr. (A) Voltage clamp recordings from a single layer V neurone. The downward defections are spontaneous sEPSCs mediated via glutamate acting at postsynaptic AMPAr. Application of 2-AP5 (50 μM) reduced the frequency of sEPSCs, but did not occlude the effect of the subsequent addition of phenytoin (50 μM), which caused a substantial further reduction. (B) Pooled data from three neurones (200 events from each neurone in each condition). The cumulative probability curve in the presence of 2-AP5 is significantly (*P* < 0.01) shifted to the right towards longer intervals (lower frequency). The addition of phenytoin caused a further significant shift (KS, *P* < 0.001). (C) In the reverse experiment, phenytoin significantly increased IEI (KS, *P* < 0.001), and 2-AP5 caused a further significant shift to the right (KS, *P* < 0.001). Pooled data from four neurones.

In three neurones, perfusion with 2-AP5 (50 μM) increased the mean IEI of sEPSCs from 211 ± 66 to 327 ± 117 ms (reflecting a decrease in frequency from 6.5 ± 2.9 to 4.2 ± 1.7 Hz, *P* < 0.01), with no significant change in amplitude (10.2 ± 1.0 pA versus 12.2 ± 1.7 pA). The decrease in frequency without change in amplitude or kinetics (see below) is strongly indicative of a presynaptic effect, and, as we have previously demonstrated, reflects blockade of the tonic facilitation of glutamate release via the presynaptic NMDA autoreceptor (Berretta and Jones, 1996a; Woodhall et al., 2001). In the same neurones, subsequent addition of phenytoin (50 μM in the presence of 2-AP5) caused a further increase in IEI to 459 ± 175 ms (3.4 ± 1.6 Hz, *P* < 0.05), again without change in amplitude. Fig. 2A shows examples of voltage clamp recordings from one neurone, and cumulative probability analysis of IEI in pooled data from all three neurones (Fig. 2B). There was a shift towards longer intervals in the presence of 2-AP5, and a further shift to the right with the addition of phenytoin. On average, the frequency of sEPSCs was reduced by −37 ± 8% by 2-AP5. With the addition of the anticonvulsant the total reduction compared to control was −55 ± 3%. Comparing the frequency of events in phenytoin plus 2-AP5 to 2-AP5 alone gave an average reduction of −21 ± 4%. sEPSC rise times (10–90%) were 2.0 ± 0.4 ms in control, 2.1 ± 0.2 ms in the presence of 2-AP5, and 2.6 ± 0.6 ms with subsequent addition of phenytoin. Corresponding times to 60% decay were 5.1 ± 2.0, 5.6 ± 2.3 and 5.4 ± 1.4 ms, respectively.

We conducted the reverse experiment in a further four neurones. Phenytoin, applied alone, increased IEI from 163 ± 36 to 250 ± 43 ms, and subsequent perfusion with 2-AP5 resulted in a further increase in IEI to 630 ± 258 ms. The respective changes in sEPSC frequency were from 7.8 ± 2.5 to 4.6 ± 1.1 (*P* = 0.05) with phenytoin, and to 2.4 ± 0.8% (*P* < 0.01) on addition of 2-AP5. Again, there were no changes in amplitude (not shown). The pooled cumulative probability analysis for IEI data are illustrated in Fig. 2C, showing clearly the shift towards longer intervals in phenytoin, and a further shift when 2-AP5 was added. The mean reductions in sEPSC frequency were −37 ± 4% with phenytoin alone, and −71 ± 7% when both drugs were present. This corresponds to a decrease in frequency of −47 ± 10% when comparing phenytoin alone to phenytoin plus 2-AP5.

Rise times (control 1.5 ± 0.1 ms; phenytoin 2.1 ± 0.5 ms; phenytoin plus 2-AP5 1.8 ± 0.4 ms) and decay times (5.3 ± 0.3 ms versus 5.4 ± 0.2 ms versus 5.3 ± 0.3 ms) were again unaltered.

### Felbamate

**Figure 3:**
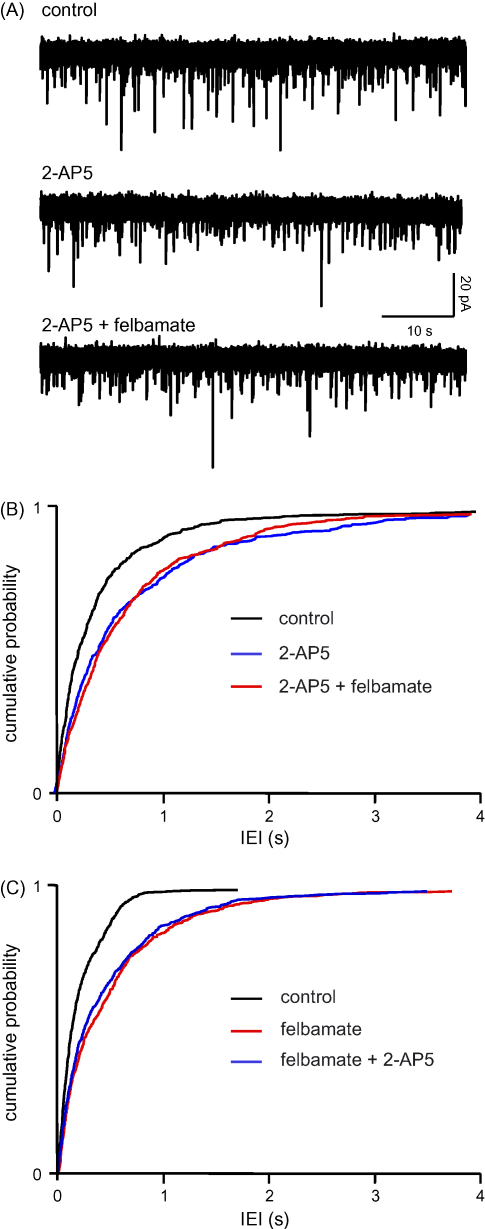
Felbamate acts via the presynaptic NMDAr. Details as in Fig. 1. (A) 2-AP5 (50 μM) reduces sEPSC frequency but the addition of felbamate (100 μM) has no further effect. (B) This is confirmed by pooled data for IEI in three neurones where there was a significant (KS, *P* < 0.001) rightward shift in the cumulative probability curve in 2-AP5, but the distribution with the addition of felbamate is completely overlapping. (C) Similar data were obtained in the reverse experiment in four neurones where felbamate occluded 2-AP5.

In three neurones 2-AP5 (50 μM) again reduced glutamate release, reflected by an increase in mean IEI (from 522 ± 154 to 950 ± 325 ms; frequency 2.1 ± 0.6 to 1.2 ± 0.3 Hz, *P* < 0.05) with no obvious change in sEPSC amplitude (13.5 ± 3.1 pA versus 12.5 ± 1 pA). However, subsequent addition of felbamate (100 μM), elicited no further change in IEI (883 ± 277 ms). In fact, in two neurones, sEPSC frequency was slightly increased when felbamate was added, although overall there was no marked change (1.3 ± 0.3 Hz, *P* > 0.1), and again amplitude remained about the same (12.6 ± 1.2 Hz). Fig. 3A shows voltage clamp recordings from one neurone and the cumulative probability analysis of pooled data are illustrated in Fig. 3B. sEPSC rise times were unaltered at 2.0 ± 0.4 ms in control, 2.4 ± 0.3 ms in the presence of 2-AP5, and 2.5 ± 0.4 ms with the addition of felbamate. Corresponding times to 60% decay were 5.3 ± 1.8, 6.0 ± 1.2 and 5.8 ± 1.9 ms, respectively.

The reverse experiment was conducted in four neurones and gave very similar results. Felbamate alone decreased EPSC frequency from 5.1 ± 1.1 to 2.5 ± 0.9 Hz (*P* < 0.01), IEI increasing from 238 ± 67 ms versus 587 ± 193 ms. Now, addition of 2-AP5 failed to cause any significant further change (2.6 ± 0.9 Hz; 528 ± 140 ms). The pooled cumulative probability data for IEI from the four neurones can be seen in Fig. 3C. Mean amplitudes were essentially the same throughout (11.2 ± 3.8 pA versus 10.7 ± 4.1 pA versus 11.2 ± 4.5 pA). Rise times were also unaltered (control 2.2 ± 0.6 ms; felbamate 2.2 ± 0.7 ms; felbamate plus 2-AP5 2.4 ± 0.6 ms), as were decay times (4.8 ± 0.6 ms versus 4.7 ± 0.7 ms versus 4.4 ± 0.5 ms.

The normalised data supported the observation that the NMDA antagonist and the anticonvulsant could occlude each other's effects. Thus, application of 2-AP5 reduced sEPSC frequency by −48 ± 3%, and the percentage reduction in the presence of both 2-AP5 and felbamate was virtually the same at −43 ± 1%. When the effect of felbamate was normalised to the frequency in 2-AP5, there was a slight increase to +10 ± 5%. Application of felbamate alone in the second set of experiments reduced frequency by −55 ± 9% and with addition of 2-AP5 the total reduction was −52 ± 8%. This reflected a change of +7 ± 12% when 2-AP5 was normalised to the frequency in felbamate alone.

### Gabapentin

**Figure 4:**
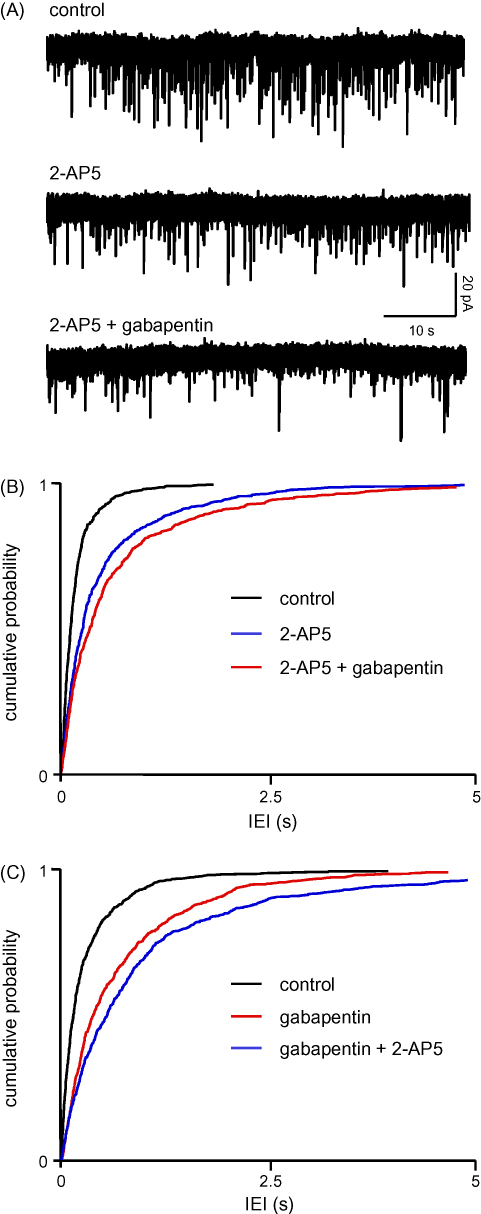
(A–C) Gabapentin does not act via presynaptic NMDAr. Details as in Fig. 2. The effect of gabapentin (25 μM) was very similar to that seen with phenytoin (Fig. 2) and differed from the occlusion seen with felbamate (Fig. 3).

Gabapentin has been suggested to target presynaptic NMDAr (Suarez et al., 2005), so we compared its effects to those of the other anticonvulsants. sEPSC frequency in three neurones was significantly (*P* < 0.05) reduced from 6.8 ± 2 to 3.1 ± 1.4 Hz (IEI: 188 ± 72 ms versus 552 ± 290 ms) during perfusion with 2-AP5. Addition of gabapentin (25 μM) caused a further reduction (*P* < 0.05) to 1.6 ± 0.4 Hz (693 ± 191 ms). Amplitudes were not significantly altered throughout (11.5 ± 2.5 pA versus 12.2 ± 4.2 pA versus 11.8 ± 4.2 pA), as were rise times (1.6 ± 0.4 ms versus 2.1 ± 0.8 ms versus 1.9 ± 0.3 ms) and decay times (5.7 ± 0.6 ms versus 6.0 ± 0.8 ms versus 5.6 ± 0.5 ms). One study is illustrated in Fig. 4A, with pooled data from the three neurones summarized in Fig. 4B. Similar results were obtained when gabapentin was applied prior to 2-AP5 (*n* = 4). In this case IEI was increased from 304 ± 85 to 712 ± 146 ms by the anticonvulsant (KS, *P* < 0.01) with a further increase to 1049 ± 397 ms when 2-AP5 was added (KS, *P* < 0.01). The results of the pooled IEI analysis are shown in Fig. 4C. As in previous studies, rise times, decay times and amplitudes were unaltered throughout (not shown).

The normalised data also indicated a lack of occlusion between the two drugs. Thus, 2-AP5 reduced sEPSC frequency by −45 ± 11% when applied alone and the total reduction with subsequent addition of gabapentin was −60 ± 17%. Comparing 2-AP5 alone to 2-AP5 plus gabapentin gave an average reduction of −30 ± 18%. In the reverse experiments, gabapentin reduced sEPSC frequency by −58 ± 5% and together with 2-AP5 the reduction was −68 ± 4%. Normalizing the 2-AP5 data to the frequency in the presence of gabapentin gave a reduction of −21 ± 13%.

## Discussion

The anticonvulsant effect of phenytoin has traditionally been associated with its ability to exert a use- and frequency-dependent block of VGSC, thereby reducing sustained repetitive firing of action potentials in cortical neurones (see Rogawski and Loscher, 2004). However, we have shown previously that phenytoin reduces the frequency but not amplitude of sEPSCs in the EC, suggesting that it also acts presynaptically to reduce glutamate release. Moreover, this effect persists in the presence of TTX, which suggests that it occurs independently of VGSC-blockade and we have suggested that it occurs downstream of Ca-entry into glutamate terminals (Cunningham et al., 2000). We have also shown that glutamate release is tonically facilitated by Ca-entry through presynaptic NMDAr in the EC (Woodhall et al., 2001), so we considered the possibility that reduction of glutamate release could occur via blockade of this receptor. The current experiments suggest that this is unlikely to be the case. The reduction in sEPSC frequency induced by phenytoin was not occluded by prior blockade of presynaptic NMDAr with 2-AP5 (nor vice versa). This argues strongly against a role of blockade of the facilitatory autoreceptor in the action of phenytoin at glutamate terminals.

Thus, the VGSC-independent mechanism of phenytoin on glutamate release therefore remains unknown. One possibility is that phenytoin interferes with the release mechanism via inhibition of PKC (see Cunningham et al., 2000). Another possibility is that it disrupts release via a direct action on the vesicles. For example, the novel anticonvulsant, levetiractem, binds specifically to the vesicle associated protein, SV2A (Lynch et al., 2004). It is conceivable that phenytoin may also act directly on the release process by altering the exocytotic process.

The present study shows that felbamate, like the other drugs we have tested previously (Cunningham et al., 2000, 2003, 2004; Cunningham and Jones, 2000), reduces the frequency, but not amplitude, of sEPSCs in EC neurones, suggesting a target on, or in, presynaptic glutamate terminals. Unlike phenytoin, however, this effect was completely occluded by pre-perfusion with 2-AP5, and the reverse experiment produced the same result. This clearly indicates that felbamate can act to block presynaptic NMDAr and thereby reduce the tonic positive feedback facilitation of glutamate release. This effect may not be completely surprising in view of the fact that felbamate has repeatedly been shown to act as an NMDAr blocker (Rho et al., 1994; Subramaniam et al., 1995; Kuo et al., 2004), as a result of either an interaction with the glycine site of the receptor (e.g. White et al., 1995; Kuo et al., 2004) or as a result of open channel blockade (Subramaniam et al., 1995; Harty and Rogawski, 2000). Whatever its action, it is significant that it has shown to be more selective for NR1-NR2B heteromeric channels (Kleckner et al., 1999; Harty and Rogawski, 2000). This would agree with our previous studies, which have shown that the presynaptic NMDAr is likely to be primarily NR2B (Woodhall et al., 2001; Yang et al., 2006).

Felbamate has also been shown to block VGCC (Stefani et al., 1996). This is unlikely to be involved in the reduction of glutamate release that we see, as it appears to be specific for L-type channels, and glutamate release at EC synapses is likely to be mediated by a combination of N, P/Q and R type channels (Cunningham et al., 2004; Woodhall et al., 2007). In addition, felbamate may also block VGSC (Taglialatela et al., 1996) leading to a reduction in sustained repetitive firing (White et al., 1992; Pisani et al., 1995), and has also been suggested to enhance GABA_A_r mediated currents (Rho et al., 1994, 1997; Kume et al., 1996). Any, or all, of these effects could be involved in the anticonvulsant actions of felbamate, but our results suggest that a reduction of the positive feedback control of glutamate release via presynaptic NMDAr needs to be included in this panoply of actions.

Suarez et al. (2005) recently suggested that presynaptic NMDAr on Schaffer-commissural fibres increase axonal excitability in CA1 as a result of increased Na-entry via the receptor. This differs from the situation in the EC where the facilitatory effect of presynaptic NMDAr on glutamate release is dependent on Ca-entry via the receptor ionophore (Woodhall et al., 2001). The present data also suggest that the ability of gabapentin to block the presynaptic NMDAr in the hippocampus is not reflected by a similar action in the EC. Thus, the reduction in glutamate release we previously reported with gabapentin (Cunningham et al., 2004) was not occluded by prior blockade of the receptor with 2-AP5 (nor vice versa). Our previous data (Cunningham et al., 2004) suggest that gabapentin may reduce glutamate release partly by blockade of Ca-entry via VGCC and partly by an unknown mechanism. The current experiments suggest that the latter is unlikely to be linked to blockade of presynaptic NMDAr. Whilst we cannot completely rule out an action of gabapentin at the facilitatory autoreceptor, it seems unlikely that this plays a major role in its anticonvulsant action.

In conclusion, we have shown that felbamate, like phenytoin, lamotrigine, gabapentin, pregabilin and valproate (Cunningham and Jones, 2000; Cunningham et al., 2000, 2003, 2004), reduces the release of glutamate at excitatory synapses in the EC. However, in contrast to at least two of these drugs, the action of felbamate seems to be largely due to blockade of presynaptic facilitatory NMDA autoreceptors. Thus, whilst a reduction in synaptic excitation may be a common underlying mechanism involved in anticonvulsant actions, different drugs may achieve this by very different means. We have recently shown that there is a developmental decline in the facilitation of glutamate release by presynaptic NMDAr in the EC, but this effect is reversed in the cortex of chronically epileptic animals (Yang et al., 2006). Thus, enhanced NMDAr mediated glutamate release could well contribute to epileptogenesis, and the ability of felbamate to block this might be a very important aspect of its anticonvulsant actions. Although the present data suggest that other drugs such as phenytoin and gabapentin do not appear to exert such an action in normal cortex, it is possible that this could change in the pathological condition. Future experiments will address this issue.
